# Testosterone Replacement Modulates Cardiac Metabolic Remodeling after Myocardial Infarction by Upregulating PPAR*α*

**DOI:** 10.1155/2016/4518754

**Published:** 2016-06-16

**Authors:** Jing Yang, Fengyue Wang, Weiju Sun, Yanli Dong, Mingyu Li, Lu Fu

**Affiliations:** ^1^Laboratory of Cardiovascular Internal Medicine Department, First Affiliated Hospital, Harbin Medical University, 23 Youzheng Street, Nangang District, Harbin, Heilongjiang 150001, China; ^2^Department of Emergency Surgery, First Affiliated Hospital, Harbin Medical University, 23 Youzheng Street, Nangang District, Harbin, Heilongjiang 150001, China

## Abstract

Despite the importance of testosterone as a metabolic hormone, its effects on myocardial metabolism in the ischemic heart remain unclear. Myocardial ischemia leads to metabolic remodeling, ultimately resulting in ATP deficiency and cardiac dysfunction. In the present study, the effects of testosterone replacement on the ischemic heart were assessed in a castrated rat myocardial infarction model established by ligating the left anterior descending coronary artery 2 weeks after castration. The results of real-time PCR and Western blot analyses showed that peroxisome proliferator-activated receptor *α* (PPAR*α*) decreased in the ischemic myocardium of castrated rats, compared with the sham-castration group, and the mRNA expression of genes involved in fatty acid metabolism (the fatty acid translocase CD36, carnitine palmitoyltransferase I, and medium-chain acyl-CoA dehydrogenase) and glucose transporter-4 also decreased. A decline in ATP levels in the castrated rats was accompanied by increased cardiomyocyte apoptosis and fibrosis and impaired cardiac function, compared with the sham-castration group, and these detrimental effects were reversed by testosterone replacement. Taken together, our findings suggest that testosterone can modulate myocardial metabolic remodeling by upregulating PPAR*α* after myocardial infarction, exerting a protective effect on cardiac function.

## 1. Introduction

Cardiac metabolic remodeling is characterized by impairments in substrate utilization and mitochondrial biogenesis and function, leading to adenosine triphosphate (ATP) deficiency [1]. Regional myocardial infarction, which induces cardiac remodeling, decreases the capacity of the heart to generate sufficient ATP to maintain cardiac function. As these metabolic changes can lead to heart failure [2], the modulation of cardiac metabolism may be an alternative approach to protect against cardiac dysfunction in myocardial infarction.

Myocardial infarction leads to partial insulin resistance accompanied by reduced fatty acid oxidation and impaired mitochondrial biogenesis in addition to the downregulation of metabolic genes [3–5]. Peroxisome proliferator-activated receptor *α* (PPAR*α*) is a nuclear receptor that functions as the primary transcriptional regulator of fatty acid metabolism in the heart. PPAR*α* target genes include fatty acid translocase (CD36) and carnitine palmitoyltransferase I (mCPT-1), which are involved in the import of fatty acids into the cell and mitochondria, and medium-chain acyl-CoA dehydrogenase (MCAD), which catalyzes the rate-limiting step in medium-chain fatty acid *β* oxidation [6]. Besides, PPAR*α* also modulates glucose metabolism. Heart-specific PPAR*α* overexpression induces the transcription of fatty acid metabolism genes and downregulates genes associated with glucose transport and PPAR*α* null mice show increased glucose transporter-4 (GLUT-4) expression and downregulation of PPAR*α* targeted genes of fatty acid metabolism [7]. PPAR*α* has emerged as an attractive target to improve metabolic remodeling.

The role of androgens in myocardial infarction is controversial. Studies showed that high levels of testosterone had adverse effects on cardiac remodeling and function after myocardial infarction [8, 9]; however, in a different study, chronic testosterone treatment had no detrimental effects after myocardial infarction and was suggested to improve long-term outcomes, reducing left ventricular end-diastolic pressure and wall stress [10]. Testosterone has also been shown to reduce the infarct size in ischemia-reperfusion of orchidectomized rats [11, 12]. In patients with coronary artery disease, testosterone deficiency is associated with poor outcomes associated with heart failure and has a significant negative impact on survival [13]. Testosterone is an important hormone that is involved in the regulation of carbohydrate, fat, and protein metabolism [14]. Low testosterone levels are associated with impaired insulin sensitivity, increased body fat percentage, truncal obesity, and dyslipidemia [15], and testosterone deficiency is a risk factor for cardiovascular morbidity and mortality among men [16]. Although testosterone has effects on cardiac metabolism [17], little is known about the role of testosterone in the regulation of cardiac metabolic remodeling in the ischemic heart. This experiment was designed to assess the effects of testosterone replacement on the cardiac metabolic remodeling via regulating the expression of PPAR*α* and its downstream genes in a castrated rat myocardial infarction model.

## 2. Materials and Methods

### 2.1. Animals

Male Wistar rats weighing 220–250 g were obtained from the Laboratory Animal Center of the First Affiliated Hospital of Harbin Medical University. The rats were maintained under temperature-controlled (22–24°C) and circadian conditions with free access to rodent chow and tap water. All experiments were performed in accordance with the protocols for the care and use of laboratory animals of the National Research Council and were approved by the ethics committee of our hospital.

### 2.2. Castration and Hormone Replacement

Rats were anesthetized with intraperitoneal injection of 10% chloral hydrate (3 mL/kg), and castration (Cas) or sham-castration (S-Cas) was randomly performed following a previously described method [18]. The animals were then randomly assigned into the four groups: (1) sham-castration+placebo (S-Cas), (2) castration+placebo (Cas), (3) castration+testosterone (Cas+T), and (4) castration+testosterone and flutamide (Cas+T+F). The different interventions were carried out according to the groupings on the same day of surgery to avoid disruption of hormonal effects [19]. Testosterone propionate (Amino Acids, P.F, Tianjin, China) dissolved in peanut oil was injected subcutaneously at a physiological dose of 2 mg/kg/d, and flutamide (Sigma Chemical Co., St. Louis, MO, USA), an antagonist of the androgen receptor (AR), dissolved in propylene glycol was injected at a dose of 30 mg/kg/d [20]. Peanut oil (2 mg/kg/d), serving as placebo, was injected into rats in groups 1 and 2.

### 2.3. Myocardial Infarction Model

Two weeks after castration, the rats received left coronary artery ligation to establish the myocardial infarction model [21]. Briefly, the rats were anesthetized with intraperitoneal injection of chloral hydrate (3 mL/kg), and then they were given mechanical positive pressure ventilation with a frequency of 65–70/min by a ventilator. The left coronary artery was ligated with 3/8 needle and 6-0 sutures. The success of establishment was confirmed by blanching of the anterior wall of the left ventricle and typical ST-segment elevation. A total of 27 rats (8 S-Cas, 6 Cas, 7 Cas+T, and 6 Cas+T+F) were included in the analysis performed 14 days after the ligation and subcutaneous injection of testosterone with/without flutamide. Additional normal rats (*n* = 8) that underwent the same procedure without occlusion were used as the control group.

### 2.4. Echocardiographic Studies

Echocardiography was performed under anesthesia at 14 days after coronary ligation. Two-dimensional and M-mode images were used to record the left ventricular end-diastolic diameter and left ventricular end-systolic diameter (LVDd and LVSd, resp.) from the parasternal long-axis views using an ultrasound machine (SONOS 7500, Philips) equipped with a 12 MHz transducer. Left ventricular ejection fraction (EF) and fractional shortening (FS) were calculated in real time. All measurements were averaged on three consecutive cardiac cycles.

Rats were euthanized, and blood samples were collected from the heart and centrifuged at 1000 ×g for 20 min to obtain serum. The hearts were excised and irrigated with saline solution. After removal of the atria, right ventricle, great vessels, and valves, the left ventricle was rapidly frozen in nitrogen and stored at −80°C or fixed in 4% paraformaldehyde and embedded in paraffin for further histological analysis.

### 2.5. Measurements of ATP

ATP concentration was measured using a kit from Jiancheng Biological Technical Institute (Nanjing, China). All procedures were performed according to the manufacturer's instructions. The peri-infarct cardiac tissues were homogenized in saline and centrifuged at 10000 ×g for 5 min. Tissue ATP was measured by spectrophotometer colorimetry at 636 nm [22].

### 2.6. Real-Time PCR

Total RNA was extracted with RNAiso Plus and reverse-transcribed to first-strand cDNA using a PrimeScript*™* RT reagent kit with gDNA Eraser (TaKaRa, Otsu, Japan) according to the manufacturer's protocol. The mRNA levels of PPAR*α*, CD36, mCPT-1, MCAD, and GLUT-4 in the peri-infarct cardiac tissues were measured by real-time PCR with SYBR Green (Roche, Germany) incorporation on an ABI 7500 Real-Time PCR System (Applied Biosystems, Foster, CA, USA). The relative quantification of gene expression was determined by comparing the target-amplified product to GAPDH, which was used as an internal standard. The primer sequences are described in Table 1.

### 2.7. Western Blotting

Proteins were extracted from the left ventricular peri-infarct tissues, separated by 10% SDS-PAGE, and transferred to polyvinylidene difluoride membranes as described previously [23]. Then, the membranes were exposed to primary antibodies against PPAR*α* (1 : 200, Santa Cruz, Dallas, Texas, USA), GLUT-4 (1 : 800, Cell Signaling Technology, Danvers, MA, USA), and GAPDH (1 : 5000, KangChen, Shanghai, China) followed by the corresponding horseradish peroxidase-conjugated secondary antibodies (1 : 2000, ZhongShan, Beijing, China). Protein bands were visualized using enhanced chemiluminescence detection reagents (Thermo Scientific*™*, Waltham, MA, USA) and exposure to X-ray film. Developed films were digitized with a scanner (Canon LiDE 110, Japan). Band intensities (area × OD) were analyzed using NIH ImageJ software (Wayne Rasband, Bethesda, MD, USA), and protein levels were normalized to GAPDH.

### 2.8. Histopathology

Myocardial samples were cut into 5 *μ*m thick cross sections along the centre of the fibrotic scar and stained with Masson-Trichrome to estimate myocardial fibrosis. The fibrotic area percent was calculated and used to quantify the degree of cardiac fibrosis in the peri-infarct region. Randomly selected digital photographs of each slice were analyzed using image analysis software (Image-Pro Plus 6.0, Media Cybernetics, Rockville, MD, USA). The percentage of fibrotic area was calculated as the ratio of positively blue-stained fibrotic area to total myocardium area.

Apoptosis was determined using the TUNEL assay as described previously [21]. The procedure was conducted by the instructions of the In Situ Cell Death Detection Kit, POD (Roche, Mannheim, Germany). The percentage of apoptotic cells was calculated from the overall number of counted cells in at least six randomly selected fields at ×400 magnification under a microscope using Image-Pro Plus 6.0 software.

### 2.9. Measurement of Serum Testosterone and Estradiol

Serum testosterone and 17*β*-estradiol levels were measured with commercially available enzyme-linked immunoassay (ELISA) kits (Uscn Life Science, Inc., Houston, TX, USA). All procedures were performed according to the manual as previously described [24]. The limit of detection was 0.0437 ng/mL for testosterone and 4.75 pg/mL for 17*β*-estradiol.

### 2.10. Statistical Analysis

Results were presented as mean ± standard deviation. One-way analysis of variance (ANOVA) followed by Tukey's post hoc test was carried out to determine differences among groups by using SPSS 20.0 statistical software (SPSS Inc., Chicago, IL, USA), and *P* < 0.05 was considered statistically significant.

## 3. Results

### 3.1. Alterations in the mRNA and Protein Expression of PPAR*α*

**Figure 1:**
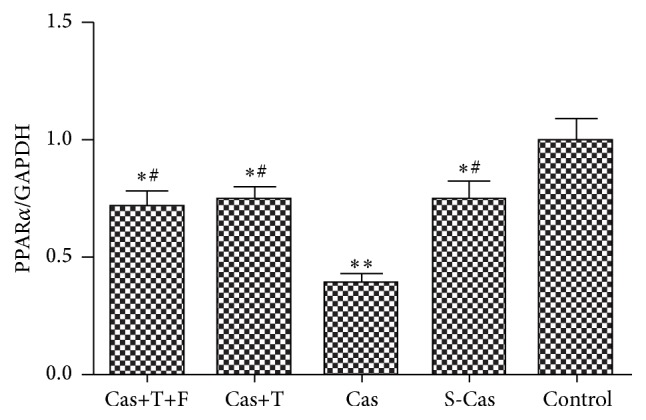
Effects of castration and testosterone replacement on the mRNA expression of PPAR*α* in heart. Values are means ± SD; *n* = 3. S-Cas: sham-castration; Cas: castration; T: testosterone; F: flutamide. ^*∗*^ *P* < 0.01 and ^*∗∗*^ *P* < 0.001 versus control group; ^#^ *P* < 0.01 versus Cas.

**Figure 2:**
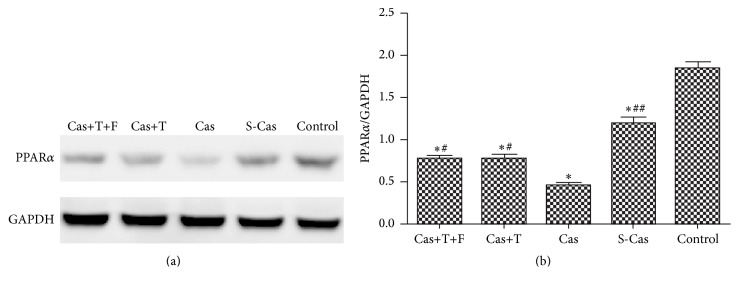
Effects of castration and testosterone replacement on the protein expression of PPAR*α* in heart. (a) Western blotting result for PPAR*α* protein level. (b) Quantitation of PPAR*α* protein level. Values are means ± SD; *n* = 3. S-Cas: sham-castration; Cas: castration; T: testosterone; F: flutamide. ^*∗*^ *P* < 0.001 versus control group; ^#^ *P* < 0.01 and ^##^ *P* < 0.001 versus Cas.

PPAR*α* plays a key role in modulating cardiac energy metabolism. In the present study, real-time PCR (Figure 1) and Western blotting (Figures 2(a) and 2(b)) showed that PPAR*α* was downregulated at the mRNA and protein levels in the S-Cas group compared with the control group (*P* < 0.01), indicating that myocardial infarction-induced metabolic remodeling involved the suppression of PPAR*α* signaling. Castration further decreased PPAR*α* expression of mRNA and protein (Cas versus S-Cas, *P* < 0.01), which was rescued by exposure to testosterone with an increase of mRNA and protein expression (Cas+T versus Cas, *P* < 0.01); additional flutamide treatment did not downregulate PPAR*α* levels compared with the group treated with testosterone alone (*P* > 0.05). Taken together, these results suggested that endogenous testosterone deprivation impaired PPAR*α* signaling in a rat model of myocardial infarction, and this effect was reversed by testosterone replacement.

### 3.2. mRNA Expression of Fatty Acid Metabolism Related Genes

**Figure 3:**
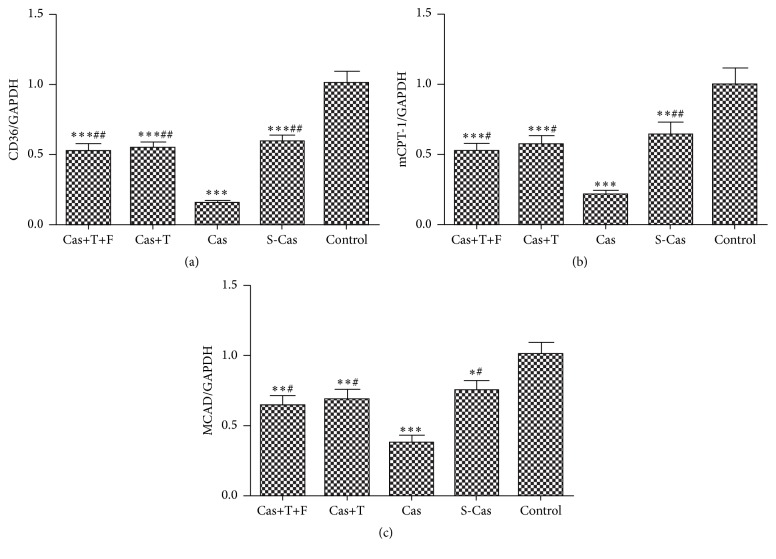
Effects of castration and testosterone replacement on the mRNA expression of fatty acid metabolism. (a–c) mRNA expression levels of CD36, mCPT-1, and MCAD, respectively. Values are means ± SD; *n* = 3. S-Cas: sham-castration; Cas: castration; T: testosterone; F: flutamide. ^*∗*^ *P* < 0.05, ^*∗∗*^ *P* < 0.01, and ^*∗∗∗*^ *P* < 0.001 versus control group; ^#^ *P* < 0.01 and ^##^ *P* < 0.001 versus Cas.

The mRNA levels of key regulators of fatty acid metabolism (CD36, mCPT-1, and MCAD) were assessed in the different groups (Figure 3). The expression of CD36, mCPT-1, and MCAD (*P* < 0.05) was downregulated in the S-Cas group compared with the control group. Castration further decreased the mRNA levels of CD36, mCPT-1, and MCAD (*P* < 0.01) compared with the S-Cas group, whereas testosterone replacement increased their expression (*P* < 0.01) compared with the castrated rats. Flutamide did not antagonize the effects of testosterone replacement on the expression of CD36, mCPT-1, and MCAD (*P* > 0.05) (Cas+T+F versus Cas+T).

### 3.3. Effects of Testosterone on the Expression of GLUT-4

**Figure 4:**
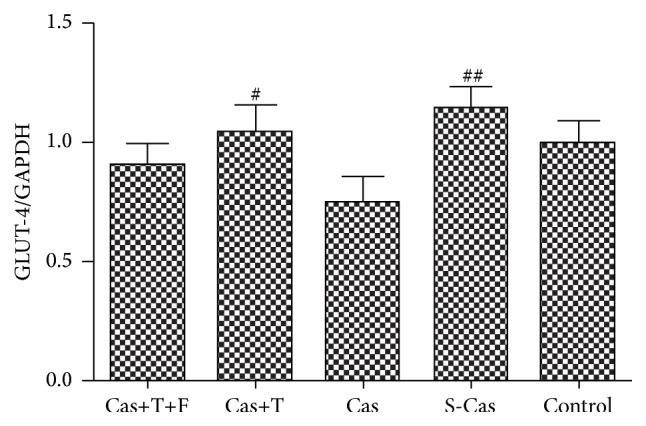
Effects of castration and testosterone on the mRNA expression of GLUT-4. Values are means ± SD; *n* = 3. S-Cas: sham-castration; Cas: castration; T: testosterone; F: flutamide. ^#^ *P* < 0.05 and ^##^ *P* < 0.01 versus Cas.

**Figure 5:**
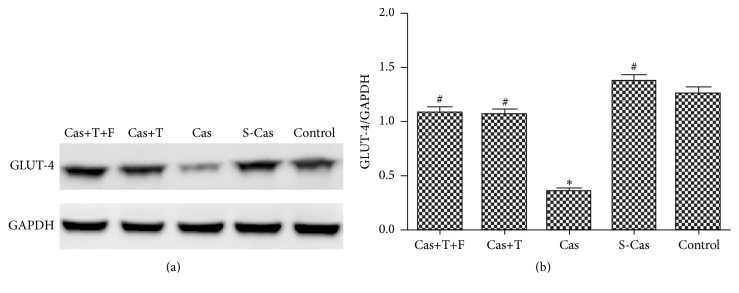
Effects of castration and testosterone on the protein expression of GLUT-4. (a) Western blotting result for GLUT-4 protein level. (b) Quantitation of GLUT-4 protein level. Values are means ± SD; *n* = 3. S-Cas: sham-castration; Cas: castration; T: testosterone; F: flutamide. ^*∗*^ *P* < 0.001 versus control group; ^#^ *P* < 0.001 versus Cas.

The mRNA and protein levels of GLUT-4 (Figures 4 and 5), which was in charge of glucose transport, were higher in the S-Cas group compared with the control group but without significant differences (*P* > 0.05). Castration decreased the mRNA and protein levels of GLUT-4 (Cas versus S-Cas, *P* < 0.01), while testosterone could attenuate the decrease in GLUT-4 compared with the castrated rats (*P* < 0.05). Flutamide did not block the effect of testosterone (Cas+T+F versus Cas+T, *P* > 0.05).

### 3.4. Changes in ATP Concentration

**Figure 6:**
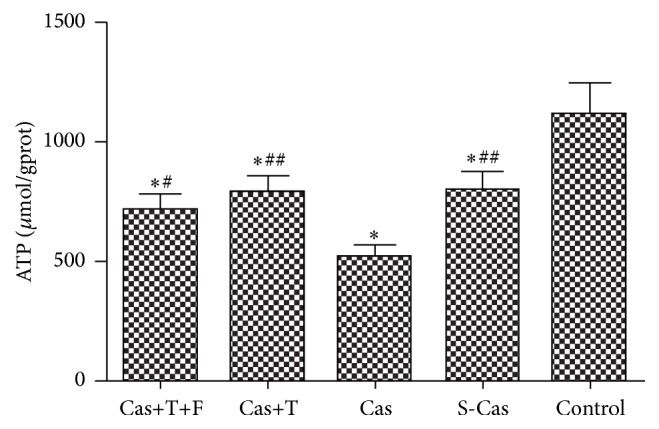
Comparison of ATP concentration in different groups. Values are means ± SD; *n* = 6. S-Cas: sham-castration; Cas: castration; T: testosterone; F: flutamide. ^*∗*^ *P* < 0.001 versus control group; ^#^ *P* < 0.01 and ^##^ *P* < 0.001 versus Cas.

The concentration of ATP in rat left ventricular tissues was compared among groups (Figure 6). ATP levels were lower in the S-Cas group than in the control group (794.80 ± 82.97 versus 1109.67 ± 140.17 *μ*mol/gprot, *P* < 0.001), and castration further decreased the levels of ATP compared with the S-Cas group (514.96 ± 56.96 versus 794.80 ± 82.97 *μ*mol/gprot, *P* < 0.001). With the treatment of testosterone, the levels of ATP were restored, compared with the castrated rats (783.81 ± 76.22 versus 514.96 ± 56.96 *μ*mol/gprot, *P* < 0.001). Additional flutamide treatment decreased ATP levels compared with the testosterone group but without statistical significance (715.04 ± 67.57 versus 783.81 ± 76.22 *μ*mol/gprot, *P* > 0.05).

### 3.5. Effects of Testosterone on Cardiac Function

**Figure 7:**
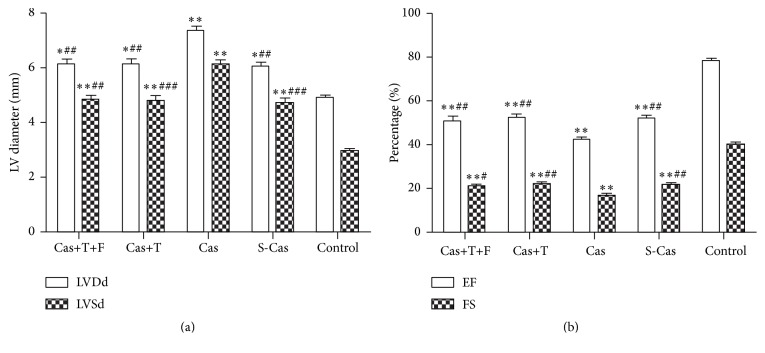
Echocardiography results 14 days after ligation. (a) Left ventricular end-diastolic diameter (LVDd) and left ventricular end-systolic diameter (LVSd) measurements for all groups. (b) Ejection fraction (EF) and fractional shortening (FS) results for all groups. Values are means ± SD; *n* = 4. S-Cas: sham-castration; Cas: castration; T: testosterone; F: flutamide. ^*∗*^ *P* < 0.01 and ^*∗∗*^ *P* < 0.001 versus control group; ^#^ *P* < 0.05, ^##^ *P* < 0.01, and ^###^ *P* < 0.001 versus Cas.

Cardiac function was evaluated by echocardiography at 14 days after ligation (Figure 7). The left ventricular end-diastolic and end-systolic diameters of myocardial infarction hearts were higher than those of the control group (*P* < 0.01), indicating cardiac dilation, whereas the EF and FS were decreased in myocardial infarction hearts (*P* < 0.001), suggesting impaired cardiac function. Castration aggravated the impairment in cardiac function, further reducing EF (42.22 ± 2.29% versus 51.98 ± 2.95%, *P* < 0.01) and FS (16.72 ± 1.09% versus 21.71 ± 1.61%, *P* < 0.01) and increasing LVDd (7.32 ± 0.35 versus 6.02 ± 0.32 mm, *P* < 0.01) and LVSd (6.10 ± 0.34 versus 4.72 ± 0.33 mm, *P* < 0.001), compared with the S-Cas group. Testosterone replacement decreased the LVDd to 6.09 ± 0.43 mm (*P* < 0.01) and LVSd to 4.76 ± 0.42 mm (*P* < 0.001) and improved myocardial performance, as indicated by the increase in EF (52.41 ± 3.00% versus 42.22 ± 2.29%, *P* < 0.01) and FS (21.95 ± 1.65% versus 16.72 ± 1.09%, *P* < 0.01) values, compared with the castrated rats. The differences in these parameters between the Cas+T+F group and the Cas+T group did not reach statistical significance (all *P* > 0.05).

### 3.6. Effects of Testosterone on Myocardial Apoptosis and Fibrosis

**Figure 8:**
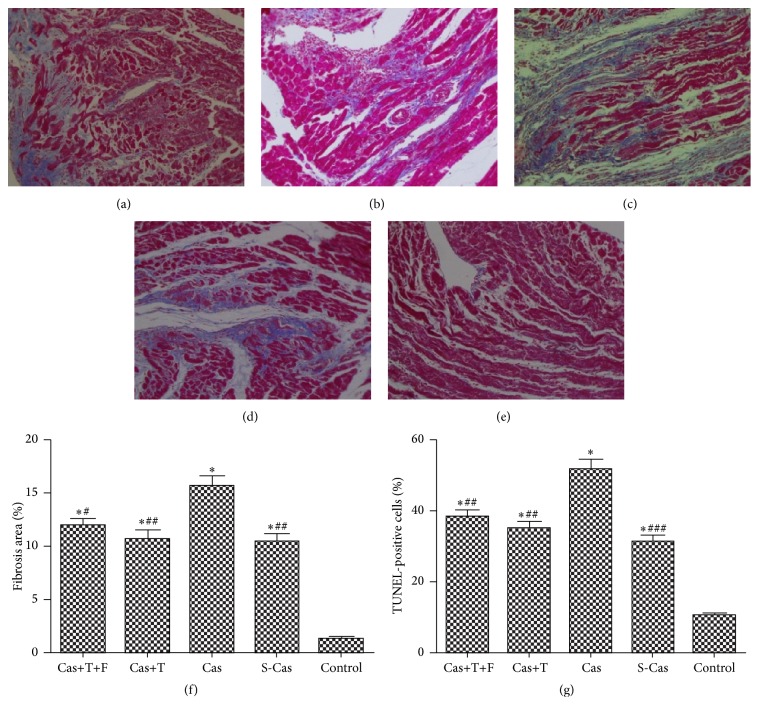
Effects of castration and testosterone replacement on myocardial fibrosis and apoptosis. Representative magnification (200x) of Masson-Trichrome sections of the heart. (a) Cas+T+F group, (b) Cas+T group, (c) Cas group, (d) S-Cas group, (e) control group, and (f) quantitative analysis of fibrotic area. (g) The TUNEL-positive cells percent expressed as a percent of normal nuclei. Values are means ± SD; *n* = 3. S-Cas: sham-castration; Cas: castration; T: testosterone; F: flutamide. ^*∗*^ *P* < 0.001 versus control group; ^#^ *P* < 0.05, ^##^ *P* < 0.01, and ^###^ *P* < 0.001 versus Cas.

The results of TUNEL staining in the different groups were shown in Figure 8(g). The number of TUNEL-positive nuclei was higher in the S-Cas group than in the control group (31.63 ± 2.29% versus 10.68 ± 0.93%, *P* < 0.001), and castration exacerbated myocardial apoptosis compared to the S-Cas group (*P* < 0.001). Testosterone treatment significantly inhibited apoptosis with a lower percent of apoptotic cells than that of the castrated group (35.10 ± 3.52% versus 51.59 ± 5.45%, *P* < 0.01).

Masson-Trichrome staining was used to estimate the degree of myocardial fibrosis of all groups in Figures 8(a)–8(f). The area of fibrosis was significantly higher in S-Cas rats than in control animals (10.29 ± 1.47% versus 1.25 ± 0.14%, *P* < 0.001), and castration aggravated myocardial fibrosis (*P* < 0.01). Testosterone replacement attenuated the degree of fibrosis to 10.72 ± 1.51%, as compared to the castrated group of 15.63 ± 1.63% (*P* < 0.01). Flutamide had no effect on myocardial apoptosis and fibrosis (*P* > 0.05). The results indicated that testosterone plays a protective effect against myocardial apoptosis and fibrosis.

### 3.7. Serum Testosterone and Estrogen Concentration

**Figure 9:**
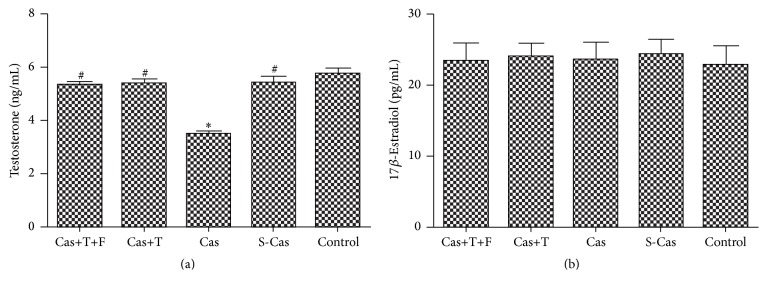
Serum testosterone levels (a) and 17*β*-estradiol levels (b). Values are means ± SD; *n* = 6. S-Cas: sham-castration; Cas: castration; T: testosterone; F: flutamide. ^*∗*^ *P* < 0.001 versus control group; ^#^ *P* < 0.001 versus Cas.

As shown in Figure 9(a), castration significantly decreased the testosterone levels (Cas versus S-Cas, *P* < 0.001), whereas testosterone replacement restored the serum levels of testosterone compared with the castrated rats (5.36 ± 0.43 versus 3.48 ± 0.25 ng/mL, *P* < 0.001). There were no statistically significant differences in serum testosterone levels between the control group, the Cas+T group, and the Cas+T+F group.

The average serum 17*β*-estradiol concentrations were comparable between the groups (Figure 9(b)). These results suggested that estrogen would exert a similar effect on each experimental group.

## 4. Discussion

In the present study, the effects of testosterone on cardiac metabolism in the ischemic heart were investigated using a rat model of myocardial infarction. Our results showed that castration decreased the levels of PPAR*α* and inhibited downstream signaling, downregulating the expression of fatty acid and glucose metabolism related genes. Castration reduced the concentration of ATP and increased cardiomyocyte apoptosis and cardiac fibrosis, aggravating cardiac dysfunction associated with myocardial infarction. Testosterone therapy reversed these unfavorable outcomes.

Alterations in cardiac metabolism play a key role in the pathogenesis and progression of myocardial ischemia and heart failure [25]. These metabolic alterations, which are termed metabolic remodeling, include a shift from fatty acids to glucose as the preferred energy substrate, decreased oxidative phosphorylation, and impaired energy transfer, leading to ATP deficiency and subsequent contractile dysfunction. Because of the close association of myocardial function with energy metabolism, metabolic pathways are potential therapeutic targets for the treatment of cardiac dysfunction [26]. During the early stages of cardiac remodeling, the myocardial energy source switches from fatty acids to glucose. A reduction in cardiac fatty acid metabolism, including the downregulation of fatty acid transporters and oxidative enzymes, has been reported in rat models of myocardial infarction-induced systolic dysfunction; however, myocardial infarction-induced alterations in cardiac glucose metabolism remain increased [3–5]. Despite the higher efficiency of glucose metabolism compared with that of fatty acids, the increase in ATP yield could not be sufficient to make up for the ATP deficiency, aggravating the progression of heart failure [27]. Lou et al. demonstrated that boosting fatty acid oxidation not glucose could enhance the energy production of infarct-remodeled rat hearts after conditioning against ischemia/reperfusion injury, which may promote postischemic contractile recovery [28]. Thus, reversal of metabolic shift may be beneficial for improving postischemic contractile dysfunction [29].

The transcription factor PPAR*α* plays an important role in the modulation of cardiac metabolism by optimizing substrate selection. Reduced activity of PPAR*α* results in downregulation of the expression of genes involved in fatty acid transport and metabolism [30]. Meanwhile, PPAR*α* KO mice exhibited reliance on glucose for cardiac ATP production with increased glucose uptake and GLUT4 expression [31, 32]. Therefore, modulation of PPAR*α* activation has been proposed as a therapeutic approach to improve myocardial function [33]. PPAR*α* is downregulated in response to cardiac hypertrophy [34], myocardial infarction [35], and heart failure [36] associated with the decrease in fatty acid utilization. The results of the present study showed that castration decreased the mRNA and protein expression of PPAR*α* in the ischemic myocardium and this effect was reversed by testosterone replacement therapy. Our results also showed that genes related to fatty acid uptake and oxidation were downregulated in myocardial infarction rats compared with control rats. The expression of fatty acid metabolism genes, including CD36, mCPT-1, and MCAD, was further downregulated by castration, whereas expression levels were restored by testosterone. Besides, testosterone replacement restored ATP levels in the castrated rat after myocardial infarction. These results suggested that testosterone could enhance fatty acid metabolism to increase ATP generation for the ischemic heart by upregulating PPAR*α*. We have demonstrated that testosterone could protect mitochondria in the postinfarct myocardium and attenuates a decrease in ATP levels and cardiomyocyte apoptosis [24]. In line with our findings, chronic activation of PPAR*α* upregulates the fatty acid metabolic pathway despite the accumulation of myocardial triglycerides without worsening left ventricular dysfunction in a rat infarct model of heart failure [37].

Although Collett et al. suggested that PPAR*α* is an androgen-negative gene in the human prostate [38], another study showed that the adrenal androgen dehydroepiandrosterone could induce peroxisome proliferative response in rats, probably by the androgen-mediated increase in PPAR*α* [39]. In the present study, we also demonstrated that testosterone could upregulate PPAR*α* expression. However, the effects of testosterone on PPAR*α* and the expression of downstream fatty acid metabolism genes could not be antagonized by flutamide. Flutamide, an antagonist of the AR, could block the effects induced by testosterone. The above-mentioned studies indicated that testosterone could modulate PPAR*α* expression through AR-independent mechanism. We have proved that testosterone can partly via the AMP-activated protein kinase- (AMPK-) peroxisome proliferator-activated receptor *γ* coactivator-1*α* (PGC-1*α*) pathway protect against mitochondrial dysfunction and cardiomyocyte apoptosis in the postinfarct myocardium [24]. PGC-1*α* can bind to the heterodimers formed by PPAR*α* and retinoic acid-activated receptor (RXR) and then coactivate PPAR*α* to enhance fatty acid utilization in myocardium [6]. Moreover, AMPK activator can upregulate PPAR*α* signaling pathway to inhibit cardiac hypertrophy [40]. However, Tennakoon et al. demonstrate that androgens regulate prostate cancer cell growth via AR-AMPK-PGC-1*α* signaling to promote mitochondrial biogenesis and induce metabolic switch [41]. Therefore, we should further investigate the interaction among AR, PPAR*α*, and RXR and elucidate the exact mechanisms of testosterone modulating metabolic remodeling in postinfarcted heart.

The effects of testosterone can be mediated by the conversion of testosterone to estrogen by the enzyme aromatase. Therefore, we measured serum estradiol concentrations to exclude its influence. The lack of differences in serum 17*β*-estradiol levels among the groups implied that the effects of estrogen would be similar in each group.

MHC-PPAR*α* mice, which are characterized by cardiac-specific PPAR*α* overexpression, show decreased glucose transport [42, 43]. However, in the present study, testosterone increased the expression of PPAR*α* as well as GLUT-4. The effect of testosterone on GLUT-4 mRNA levels may be mediated by AMPK. This was supported by a previous study showing that testosterone increased GLUT4-dependent glucose uptake, which was mediated by Ca^2+^/calmodulin protein kinase and AMPK in cultured cardiomyocytes [44]. To elucidate the mechanism by which testosterone modulates glucose metabolism in the ischemic heart, future studies will be aimed at investigating the effect of testosterone on insulin signaling and glucose oxidation, as well as other glucose metabolism pathways, such as glycolysis and the pentose phosphate pathway.

In conclusion, the present study showed that testosterone insufficiency downregulated PPAR*α* and altered the mRNA expression of fatty acid metabolism and glucose transport related genes, impairing ATP production in the ischemic myocardium. Testosterone replacement therapy reversed these unfavorable changes and improved cardiac metabolic remodeling and cardiac dysfunction. These data provide new application of testosterone in cardiovascular diseases.

## Figures and Tables

**Table 1:** Primers for real-time PCR.

	Primer
PPAR*α*	F: 5′-TTTGTGGGGCTGGAGGGTTCGTG-3′
	R: 5′-GCCACAGAGCACCAATCTGTGA-3′

CD36	F: 5′-CCTATTGGGAAAGTTATTGCG-3′
	R: 5′-GTTGTCTGGGTTCTGGAGTG-3′

mCPT-1	F: 5′-CGAGTTCAGAAACGAACGCCC-3′
	R: 5′-GTGCATGCCACCCCTTATGC-3′

MCAD	F: 5′-TGTGTGCCTACTGCGTGACA-3′
	R: 5′-TCGGCCTCCACGATGAATCC-3′

GLUT-4	F: 5′-AGGCCGGGACACTATACCCTA-3′
	R: 5′-TCTGTGGGGCGTTGATGACT-3′

GAPDH	F: 5′-GGAAAGCTGTGGCGTGAT-3′
	R: 5′-AAGGTGGAAGAATGGGAGTT-3′

## References

[1] Doenst T., Nguyen T. D., Abel E. D. (2013). Cardiac metabolism in heart failure: implications beyond ATP production. *Circulation Research*.

[2] Neubauer S. (2007). The failing heart—an engine out of fuel. *The New England Journal of Medicine*.

[3] Amorim P. A., Nguyen T. D., Shingu Y. (2010). Myocardial infarction in rats causes partial impairment in insulin response associated with reduced fatty acid oxidation and mitochondrial gene expression. *Journal of Thoracic and Cardiovascular Surgery*.

[4] Heather L. C., Cole M. A., Lygate C. A. (2006). Fatty acid transporter levels and palmitate oxidation rate correlate with ejection fraction in the infarcted rat heart. *Cardiovascular Research*.

[5] Rosenblatt-Velin N., Montessuit C., Papageorgiou I., Terrand J., Lerch R. (2001). Postinfarction heart failure in rats is associated with upregulation of GLUT-1 and downregulation of genes of fatty acid metabolism. *Cardiovascular Research*.

[6] Rowe G. C., Jiang A., Arany Z. (2010). PGC-1 coactivators in cardiac development and disease. *Circulation Research*.

[7] Finck B. N. (2007). The PPAR regulatory system in cardiac physiology and disease. *Cardiovascular Research*.

[8] Cavasin M. A., Tao Z.-Y., Yu A.-L., Yang X.-P. (2006). Testosterone enhances early cardiac remodeling after myocardial infarction, causing rupture and degrading cardiac function. *American Journal of Physiology—Heart and Circulatory Physiology*.

[9] Cavasin M. A., Sankey S. S., Yu A.-L., Menon S., Yang X.-P. (2003). Estrogen and testosterone have opposing effects on chronic cardiac remodeling and function in mice with myocardial infarction. *American Journal of Physiology—Heart and Circulatory Physiology*.

[10] Nahrendorf M., Frantz S., Hu K. (2003). Effect of testosterone on post-myocardial infarction remodeling and function. *Cardiovascular Research*.

[11] Pongkan W., Chattipakorn S. C., Chattipakorn N. (2015). Chronic testosterone replacement exerts cardioprotection against cardiac ischemia-reperfusion injury by attenuating mitochondrial dysfunction in testosterone-deprived rats. *PLoS ONE*.

[12] Tsang S., Wu S., Liu J., Wong T. M. (2008). Testosterone protects rat hearts against ischaemic insults by enhancing the effects of *α*
_1_-adrenoceptor stimulation. *British Journal of Pharmacology*.

[13] Malkin C. J., Pugh P. J., Morris P. D., Asif S., Jones T. H., Channer K. S. (2010). Low serum testosterone and increased mortality in men with coronary heart disease. *Heart*.

[14] Kelly D. M., Jones T. H. (2013). Testosterone: a metabolic hormone in health and disease. *Journal of Endocrinology*.

[15] Wang C., Jackson G., Jones T. H. (2011). Low testosterone associated with obesity and the metabolic syndrome contributes to sexual dysfunction and cardiovascular disease risk in men with type 2 diabetes. *Diabetes Care*.

[16] Haring R., John U., Völzke H. (2012). Low testosterone concentrations in men contribute to the gender gap in cardiovascular morbidity and mortality. *Gender Medicine*.

[17] Wittnich C., Tan L., Wallen J., Belanger M. (2013). Sex differences in myocardial metabolism and cardiac function: an emerging concept. *Pflugers Archiv European Journal of Physiology*.

[18] Golden K. L., Marsh J. D., Jiang Y., Brown T., Moulden J. (2003). Gonadectomy of adult male rats reduces contractility of isolated cardiac myocytes. *American Journal of Physiology—Endocrinology and Metabolism*.

[19] Maddali K. K., Korzick D. H., Tharp D. L., Bowles D. K. (2005). PKC*δ* mediates testosterone-induced increases in coronary smooth muscle Cav1.2. *The Journal of Biological Chemistry*.

[20] Sun J., Fu L., Tang X. (2011). Testosterone modulation of cardiac *β*-adrenergic signals in a rat model of heart failure. *General and Comparative Endocrinology*.

[21] Chen Y., Fu L., Han Y. (2012). Testosterone replacement therapy promotes angiogenesis after acute myocardial infarction by enhancing expression of cytokines HIF-1a, SDF-1a and VEGF. *European Journal of Pharmacology*.

[22] Liu Y., Geng J., Liu Y. (2013). *β*3-Adrenoceptor mediates metabolic protein remodeling in a rabbit model of tachypacing-induced atrial fibrillation. *Cellular Physiology and Biochemistry*.

[23] Han Y., Fu L., Sun W. (2009). Neuroprotective effects of testosterone upon cardiac sympathetic function in rats with induced heart failure. *European Journal of Pharmacology*.

[24] Wang F., Yang J., Sun J. (2015). Testosterone replacement attenuates mitochondrial damage in a rat model of myocardial infarction. *Journal of Endocrinology*.

[25] Abozguia K., Shivu G. N., Ahmed I., Phan T. T., Frenneaux M. P. (2009). The heart metabolism: pathophysiological aspects in ischaemia and heart failure. *Current Pharmaceutical Design*.

[26] Azevedo P. S., Minicucci M. F., Santos P. P., Paiva S. A. R., Zornoff L. A. M. (2013). Energy metabolism in cardiac remodeling and heart failure. *Cardiology in Review*.

[27] Van Bilsen M., Van Nieuwenhoven F. A., Van Der Vusse G. J. (2009). Metabolic remodelling of the failing heart: beneficial or detrimental?. *Cardiovascular Research*.

[28] Lou P.-H., Zhang L. Y., Lucchinetti E. (2013). Infarct-remodelled hearts with limited oxidative capacity boost fatty acid oxidation after conditioning against ischaemia/reperfusion injury. *Cardiovascular Research*.

[29] Tanno M., Kuno A. (2013). Reversal of metabolic shift in post-infarct-remodelled hearts: possible novel therapeutic approach. *Cardiovascular Research*.

[30] Burkart E. M., Sambandam N., Han X. (2007). Nuclear receptors PPAR*β*/*δ* and PPAR*α* direct distinct metabolic regulatory programs in the mouse heart. *Journal of Clinical Investigation*.

[31] Campbell F. M., Kozak R., Wagner A. (2002). A role for peroxisome proliferator-activated receptor *α* (PPAR*α*) in the control of cardiac malonyl-CoA levels: reduced fatty acid oxidation rates and increased glucose oxidation rates in the hearts of mice lacking PPAR*α* are associated with higher concentrations of malonyl-CoA and reduced expression of malonyl-CoA decarboxylase. *The Journal of Biological Chemistry*.

[32] Panagia M., Gibbons G. F., Radda G. K., Clarke K. (2005). PPAR-*α* activation required for decreased glucose uptake and increased susceptibility to injury during ischemia. *American Journal of Physiology—Heart and Circulatory Physiology*.

[33] Pol C. J., Lieu M., Drosatos K. (2015). PPARs: protectors or opponents of myocardial function?. *PPAR Research*.

[34] Barger P. M., Brandt J. M., Leone T. C., Weinheimer C. J., Kelly D. P. (2000). Deactivation of peroxisome proliferator-activated receptor-*α* during cardiac hypertrophic growth. *The Journal of Clinical Investigation*.

[35] Masamura K., Tanaka N., Yoshida M. (2003). Myocardial metabolic regulation through peroxisome proliferator-activated receptor alpha after myocardial infarction. *Experimental and Clinical Cardiology*.

[36] Karbowska J., Kochan Z., Smolenski R. T. (2003). Peroxisome proliferator-activated receptor *α* is downregulated in the failing human heart. *Cellular and Molecular Biology Letters*.

[37] Morgan E. E., Rennison J. H., Young M. E. (2006). Effects of chronic activation of peroxisome proliferator-activated receptor-*α* or high-fat feeding in a rat infarct model of heart failure. *American Journal of Physiology—Heart and Circulatory Physiology*.

[38] Collett G. P., Betts A. M., Johnson M. I. (2000). Peroxisome proliferator-activated receptor *α* is an androgen-responsive gene in human prostate and is highly expressed in prostatic adenocarcinoma. *Clinical Cancer Research*.

[39] Hayashi F., Tamura H., Yamada J., Kasai H., Suga T. (1994). Characteristics of the hepatocarcinogenesis caused by dehydroepiandrosterone, a peroxisome proliferator, in male F-344 rats. *Carcinogenesis*.

[40] Meng R.-S., Pei Z.-H., Yin R. (2009). Adenosine monophosphate-activated protein kinase inhibits cardiac hypertrophy through reactivating peroxisome proliferator-activated receptor-*α* signaling pathway. *European Journal of Pharmacology*.

[41] Tennakoon J. B., Shi Y., Han J. J. (2014). Androgens regulate prostate cancer cell growth via an AMPK-PGC-1*α*-mediated metabolic switch. *Oncogene*.

[42] Finck B. N., Lehman J. J., Leone T. C. (2002). The cardiac phenotype induced by PPAR*α* overexpression mimics that caused by diabetes mellitus. *The Journal of Clinical Investigation*.

[43] Park S.-Y., Cho Y.-R., Finck B. N. (2005). Cardiac-specific overexpression of peroxisome proliferator–activated receptor-*α* causes insulin resistance in heart and liver. *Diabetes*.

[44] Wilson C., Contreras-Ferrat A., Venegas N. (2013). Testosterone increases GLUT4-Dependent glucose uptake in cardiomyocytes. *Journal of Cellular Physiology*.

