# Expression of Concern on “Testosterone Replacement Modulates Cardiac Metabolic Remodeling after Myocardial Infarction by Upregulating PPAR*α*”

**DOI:** 10.1155/2019/5137020

**Published:** 2019-06-11

**Authors:** PPAR Research

*PPAR Research *would like to express concern with the article titled “Testosterone Replacement Modulates Cardiac Metabolic Remodeling after Myocardial Infarction by Upregulating PPAR*α*” [1] published in* PPAR Research *in June 2016 due to flaws found in the quality of the article.

The Editorial Board believed that the experimental design is not solid enough as the control groups are missing. They thought that it is confusing that most of the data sets are labeled with n=3 in the legends which is extremely low for animal experiments. In addition, the board found that the authors described each group number in the methods which is not n=3 but slightly higher. There is no explanation why not all animals were included in all analyses and how a selection of data sets was performed. In some data sets, the authors use n=6 but some animal data sets are missing.

The authors explained that, in the Materials and Methods, additional normal rats that underwent the same procedure without LDA occlusion were used as the control group. Moreover, the number of animals which were alive after the myocardial infarction was mentioned in the Materials and Methods and the number of animals in figure legends was less than that in the Materials and Methods as the remaining alive rats were divided into several parts for different experiments. They added that, during echocardiographic measurement, two rats of Cas group died from anesthesia, so they took four rats from different groups to evaluate cardiac indexes. After echocardiographic studies, the rats were immediately executed in addition to collecting blood and heart samples. The minimum number of different groups was 6 (8 S-Cas, 6 Cas, 7 Cas+T, and 6 Cas+T+F). Accordingly, they measured ATP content and sexual hormones of six rats. Three rats were performed by histological analysis, and the other three were performed by western blotting and real-time PCR.

The Editorial Board was concerned about the death of several animals which should be clearly defined by the authors as they should state why the n was 3-6 when each treatment is mentioned with an n = 8. Moreover, the board found that the statistical analysis was not done properly and they recommended repeating the study to increase the sample size.
